# A Two-Dimensional Affinity Capture and Separation Mini-Platform for the Isolation, Enrichment, and Quantification of Biomarkers and Its Potential Use for Liquid Biopsy

**DOI:** 10.3390/biomedicines8080255

**Published:** 2020-07-30

**Authors:** Norberto A. Guzman, Daniel E. Guzman

**Affiliations:** 1 Princeton Biochemicals, Inc., Princeton, NJ 08816, USA; 2 Department of Internal Medicine, University of California at San Francisco, San Francisco, CA 94143, USA; daniel.guzman@ucsf.edu or

**Keywords:** low abundance biomarkers, frequent diagnosis-prognosis testing, circulating cells, extracellular vesicles, exosomes, liquid biopsy, immunoaffinity capillary electrophoresis, molecular biorecognition, non-coding RNAs, point-of-care instrument, proteomics, telemedicine

## Abstract

Biomarker detection for disease diagnosis, prognosis, and therapeutic response is becoming increasingly reliable and accessible. Particularly, the identification of circulating cell-free chemical and biochemical substances, cellular and subcellular entities, and extracellular vesicles has demonstrated promising applications in understanding the physiologic and pathologic conditions of an individual. Traditionally, tissue biopsy has been the gold standard for the diagnosis of many diseases, especially cancer. More recently, liquid biopsy for biomarker detection has emerged as a non-invasive or minimally invasive and less costly method for diagnosis of both cancerous and non-cancerous diseases, while also offering information on the progression or improvement of disease. Unfortunately, the standardization of analytical methods to isolate and quantify circulating cells and extracellular vesicles, as well as their extracted biochemical constituents, is still cumbersome, time-consuming, and expensive. To address these limitations, we have developed a prototype of a portable, miniaturized instrument that uses immunoaffinity capillary electrophoresis (IACE) to isolate, concentrate, and analyze cell-free biomarkers and/or tissue or cell extracts present in biological fluids. Isolation and concentration of analytes is accomplished through binding to one or more biorecognition affinity ligands immobilized to a solid support, while separation and analysis are achieved by high-resolution capillary electrophoresis (CE) coupled to one or more detectors. When compared to other existing methods, the process of this affinity capture, enrichment, release, and separation of one or a panel of biomarkers can be carried out on-line with the advantages of being rapid, automated, and cost-effective. Additionally, it has the potential to demonstrate high analytical sensitivity, specificity, and selectivity. As the potential of liquid biopsy grows, so too does the demand for technical advances. In this review, we therefore discuss applications and limitations of liquid biopsy and hope to introduce the idea that our affinity capture-separation device could be used as a form of point-of-care (POC) diagnostic technology to isolate, concentrate, and analyze circulating cells, extracellular vesicles, and viruses.

## 1. Introduction

Biomarkers are measurable substances or characteristics in the human body that act as indicators of normal biological processes, pathogenic processes, or responses to an exposure or intervention, including therapeutic interventions [1,2,3,4]. In practice, biomarkers include tools and technologies that have been used in clinical medicine for decades, and they play a critical role in all aspects of prevention, diagnosis, and treatment of disease [2]. Despite advances in laboratory technology and the availability of significant information on biomarkers, we are quite far from widespread clinical use of biomarkers due to several limitations. The slow, arduous, and challenging process by which biomarkers are translated into clinical use, makes them suffer from low sensitivity, specificity, and predictive value, particularly when they are applied to rare diseases in population screening programs [3]. As a consequence, medicine in general, has long been criticized for lagging behind other industries in both innovation and adoption [4]. However, with a new wealth of biomarker information obtained over the last decade and advancements in systems medicine, there are opportunities to make significant improvements to diagnostics and therapeutics in medicine [5].

In this manuscript, we describe a point-of-care biomarker analyzer that has been demonstrated to identify and quantify circulating cell-free small molecules and complex biomolecules in biological fluid samples by utilizing an affinity capture-separation technology. Furthermore, we will discuss the potential of this instrument to serve as a tool for detecting and analyzing cellular and subcellular entities, vesicular entities, viruses and their respective cargo contents. The ultimate goal of this biomarker analyzer is to demonstrate the potential to non-invasively and rapidly yield highly sensitive and selective tests to aid real-time clinical practice.

## 2. Definitions

The existence of different meanings for certain terminologies in clinical diagnostic chemistry and analytical chemistry is a source of confusion related to several laboratory assays, particularly immunoassays. In clinical diagnostic chemistry, a sensitive test refers to a test that will correctly identify almost all individuals who likely have a disease, and that will rarely yield a false-negative result. A specific test refers to a test that will almost always correctly rule out those who do not have a disease and will rarely yield a false-positive result [6]. In analytical chemistry, sensitivity is the minimum detectable concentration of an analyte, expressed as the limit of detection (LoD), that can be accurately measured. Specificity is the ability to accurately assess a single analyte in the presence of components which may be expected to be present in the sample matrix, such as interferences. Similar to specificity, selectivity is the ability to accurately separate out and assess multiple components present in a sample mixture or matrix [7].

Biopsy is another term that historically has referred to an invasive examination of tissue removed from a living body to discover the presence, cause, or extent of a disease [8]. The term biopsy has evolved to include both tissue biopsy and liquid biopsy, a non-invasive or a minimally invasive procedure used for the detection and isolation of circulating tumor cells, circulating tumor DNA, and exosomes circulating in biological fluids [9,10]. In turn, liquid biopsy has allowed for further prognostication and evaluation of therapeutic response in patients with malignancy. Recent studies have shown that liquid biopsy has applications in non-cancerous diseases, allowing for the examination of cells, subcellular entities or vesicles, and their chemical and biochemical contents to discover the presence, cause, or extent of any disease [11,12]. For example, the use of liquid biopsy via a high-throughput metagenomic sequencing assay has recently demonstrated both detection of a diverse array of bacterial and viral pathogens and quantification of damage to host tissues. The assay implements whole-genome sequencing of cell-free DNA (cfDNA), small fragments of DNA released by host or microbial cells into blood, urine, and other body fluids [13]. Similarly, proteins derived from the novel coronavirus 2 (SARS-CoV-2) have been analyzed by high-performance liquid chromatography (HPLC) coupled to mass spectrometry (MS) from samples obtained from coronavirus disease (COVID-19) patients [14]. We will therefore also discuss further applications of liquid biopsy in this review and its relationship to our aforementioned IACE-POC biomarker analyzer instrument.

## 3. Brief History of Biomarker-Assay Technologies

Over the years, immunoassays have been one of the main analytical tools used for the detection and quantification of simple and complex biomarkers in a wide range of biological matrices [15]. While these assays have been used in many areas of life science research, they are particularly useful in fields like clinical chemistry and food science. The success of these tests within these fields has encouraged further research to increase production of assays that provide improved diagnostic and analytical sensitivity, specificity, selectivity, and robustness. What was crucial to this success was a paradigm shift provided by three major immunoassay developments of the 20th century. These novel discoveries, dramatically changed the way analytes were detected and quantified in the laboratory, and they have been invaluable tools since (a) the radioimmunoassay by Yalow and Berson in 1960 [16]; (b) the enzyme-linked immunosorbent assay (ELISA) by Engvall and Perlmann in 1971 [17]; and (c) the production of monoclonal antibodies from mouse hybridoma cells by Kohler and Milstein in 1975 [18]. These developments have actually marked a revolution in the analysis of biomarkers, providing new insights into immunology and eventually having an impact on virtually every field of biomedical and food science investigation [15,19,20]. To date, numerous variations to the original immunoassays have been developed, including fluorescence immunoassay, chemiluminescence immunoassay, time-resolved immunoassay, lateral flow assay, etc. These immunoassays have been widely used not only in clinical chemistry and food science, but also for the detection of analytes derived from forensic samples, environmental samples, plastic products, cosmetic products, drinking water, and others [21,22,23]. Immunoassays in general are now the gold standard for biomarker detection and quantification in many laboratories around the world, due to their simplicity and user-friendly operation, relatively low cost, scalability, and utilization of small sample and reagent volumes [15,19,21,22,24]. Unfortunately, there are inherent limitations to immunoassay technologies that affect their clinical applications, such as antigen-antibody cross-reactivity, narrow concentration range of the analyte, variable reproducibility amongst different laboratories, lot-to-lot variability, and sample preparation time [24,25,26,27,28]. Additionally, complementary analytical techniques, such as gas chromatography (GC), HPLC, and supercritical fluid chromatography (SFC) coupled to various detection systems, MS, have also been used for the separation, detection, quantification, and characterization of numerous substances for specific applications [29]. However, these aforementioned techniques are costly and must be operated by skilled professionals, thus limiting the settings in which they can be used.

Although the combination of immunoassays and complementary analytical techniques is quite useful for the determination of biomarkers, each technology still has its unique advantages and disadvantages that must be considered [30]. For example, when comparing ELISA, an immunoassay, to HPLC, an analytical separation technique, ELISA is more applicable for screening purposes [31], while HPLC and HPLC-MS are used to separate, identify, and quantify individual components in a mixture. Used separately, they can generate variations in the results they produce [32]. Conversely, the merging of solid-phase extraction (SPE)/affinity-capture with chromatographic or electrophoretic methods, allows for isolation, concentration, separation, detection, quantification, and characterization of individual target biomarkers and/or structurally related analytes [25,33,34]. Moreover, coupling on-line solid-phase extraction methods to HPLC or CE instruments have been shown to provide many advantages, including speed of analysis and reproducibility of results, particularly, when antibodies, aptamers, and/or lectins have been used for the isolation and concentration of target analytes. The superior resolution power of CE permits the separation of closely structurally related analytes. Significant improvements in the analysis of biomarkers can also be achieved when powerful detectors, such as laser-induced fluorescence detection and/or mass spectrometry, are used for their detection and characterization [25,34].

## 4. Miniaturized Biomarker Analyzers

The sophistication of diagnostic techniques has come a long way since the 20th century and continues to develop rapidly [35,36]. As the clinical diagnostic laboratory has entered the 21st century, instruments have become more compact and automated, while a wide range of new cellular and molecular biomarkers have been discovered. These advancements are providing more information about the human condition quicker and more accurately than ever before [36].

During the last 50 years, the detection of biomarkers has been carried out primarily in centralized laboratories which, in most cases, use sophisticated instrumentation that may need large spaces, availability of various reagents, and highly trained personnel to operate appropriately. However, as efforts to advance healthcare delivery continue to develop, there is a need for healthcare to be less fragmented and more patient-centered [37]. One way of accomplishing this task, is through improvements in diagnostic devices: they are being reduced both in size and complexity, thereby lowering cost and sample size, while maintaining and potentially improving safety, rapidity, sensitivity, and specificity. As a result, POC instruments have been developed to be as simple and accessible as possible, allowing even a patient to self-sample biological fluids from home (e.g., glucometer) [37,38,39]. As centralized testing can be an arduous and expensive process, POC testing may therefore be one approach to facilitate diagnosis, in particular for those patients who have limited or no access to healthcare, for testing centers that have limited resources and personnel, and for those who suffer from chronic diseases that require frequent biomarker monitoring. Moreover, despite the prevalence of high-burden acute and chronic disease globally, there are relatively few diagnostic tools that have been designed for biomarker analysis in developing countries [40] and rural areas within developed countries [41].

Although point-of-care instruments have the potential to address several needs within medical diagnostics, it is of the utmost importance that the results obtained are highly sensitive and specific. Test results which provide false negative or false positive data will lead to misdiagnosis, inappropriate treatment or lack thereof, and sunken costs for healthcare systems [42]. The central role of clinical diagnostics is often underappreciated because the impact on patient care is not as readily apparent as medical intervention. Without accurate diagnosis, appropriate treatment is not possible [42].

## 5. Role of Capillary Electrophoresis in Point-of-Care Diagnostics

Though the concept of a miniaturized or personal laboratory has been voiced for several years [25,43,44,45], the creation of a modular design that is manufactured with compact and low-weight components for easy portability has proved to be difficult. Capillary electrophoresis is a separation technique that has the potential to address this limitation as it utilizes miniaturized components, including a small power supply and detectors that can fit into a small space, thereby facilitating the manufacture of an instrument no larger than the size of a lap-top computer or smaller device [25,45,46]. Instruments developed for CE use are manufactured in two formats: a conventional platform using fused-silica capillaries, and a microfluidic chip format consisting of microchannels etched or molded into a material made of glass, silicon, or a polymer such as polydimethylsiloxane [25,43,44,45,46,47,48,49,50,51,52,53,54,55,56,57,58]. Newer microfabrication techniques in particular are advancing, and microchip production using 3D is now under development [52,53]. Overall, CE offers advantages such as reduced sample and reagent consumption, lower operating costs, shorter reaction time, better portability, higher number of theoretical plates, and higher reliability [25,47,54,55,56,57,58,59,60,61,62,63,64,65,66]. Despite many advances in the CE technology, there is still a limited number of medical applications that are routinely performed in a clinical laboratory.

Before it can replace existing technologies currently used in clinical laboratories, CE has its own limitations that must be addressed. For example, the internal diameters of the capillaries and micro-channels utilized in CE are usually less than 200 micrometers, allowing the utilization of small volumes of fluids which are normally in the order of nanoliters and picoliters. Consequently, the limitations of introducing small volumes of sample into the capillary column or channel results in poor concentration limits of detection, and hence risks overshadowing the many benefits of the CE technology [25,47,61,62,63,64,65,66]. This is particularly concerning given that crucial biomarkers found in biological fluids are present in low concentrations which, if not detected and quantified, may lead to missing an important diagnosis. A misdiagnosis or delayed diagnosis further leads to incorrect treatment, delayed treatment, or no treatment at all, thereby worsening a patient’s medical condition. Unfortunately, most examples reported in the literature for the CE quantification of analytes in complex matrices are usually developed for substances found at large concentrations. While not all diagnostic errors are secondary to laboratory errors, the National Academy of Medicine estimates that all patients will experience one serious diagnostic error during their lifetime, and diagnostic errors are now the leading cause of medical malpractice claims [67].

Overcoming the poor analytical sensitivity of CE has therefore become the emphasis of many investigations related to life science applications. Promisingly, efforts to improve the analytical sensitivity of CE thus far have led to successful improvements in the limits of detection of substances found at low concentrations. Some of these methods include field-amplified sample stacking, large-volume sample stacking, pH-mediated sample stacking, on-column isotachophoresis, chromatographic preconcentration, sample stacking for micellar electrokinetic chromatography, sweeping, and derivation of analytes with fluorescent chromophores [25,47,54,55,61,62,63,64,65,66,68,69,70,71,72,73,74,75,76,77,78,79,80]. One technology in particular is the combination of solid-phase extraction methods, using affinity-based ligands, with CE. Highly selective affinity ligands or adsorbents, such as antibodies, antibody fragments, lectins, aptamers, enzymes, metal-organic framework-based affinity sorbents, and other specially designed ligands are the cornerstone for the isolation and purification of many substances and cellular-subcellular entities found in complex matrices [25,47,54,55,56,57,58,75,81,82,83,84,85,86,87,88,89,90,91,92,93,94,95,96,97,98].

Immunoaffinity capillary electrophoresis (IACE) is a disruptive technique, which combines the use of antibodies and/or other affinity ligands (e.g., lectins, aptamers, metal-organic, or others) as highly selective capture agents with the superior resolving power of capillary electrophoresis. The high-resolution analytical separation ability of IACE provides the advantage of characterizing and discerning different forms of the same protein (proteoforms) biomarker and drug metabolites, which can improve insights into disease diagnosis, monitor drug efficacy, and shorten the length of clinical trials. Since the introduction of IACE in the early 1990s by our laboratory, Terry Phillips’ laboratory, and others, the technology has increased significantly in popularity, and many applications have been reported using both conventional CE and microchip CE [25,54,55,58,61,62,63,64,66,75,88,89,90,91,92,93,94,95,96,97,98]. Furthermore, if IACE is coupled to powerful detectors such as laser-induced fluorescence detectors or mass spectrometers, a significant improvement in limits of detection can been achieved [25,75,99,100,101,102,103,104,105,106,107,108].

Since its inception, IACE technology has used a “linear or unidirectional” protocol for the introduction of samples and buffers [88,89,90,109,110,111,112,113]. A small area of the capillary known as the “analyte concentrator-microreactor” (ACM) zone or device contains affinity ligands immobilized either to a matrix localized within the cavity of the ACM device, or directly to the inner wall of a capillary or channel positioned near its inlet side. The term “analyte concentrator” refers to the function of isolating and concentrating a target analyte, usually present in a complex matrix, by using a “chemical or biochemical magnet” [25,75,88,89,90,109]. The term “microreactor”, on the other hand, refers to the function of performing a chemical, biochemical and/or cellular-organoid reaction, such as derivatization, chemical synthesis, enzymatic reaction, pharmacokinetic studies, or therapy response-prediction [25,75,112,113,114,115,116,117,118,119,120,121,122]. The linear or unidirectional IACE capture-separation system aimed at performing on-line concentration and separation presents two problems: backpressure and contamination of the surface of the separation capillary or channel after repetitive uses. The backpressure is formed when there is use of a beaded matrix requiring porous frit structures, or when there is a compacted continuous bed composed of a porous micro/nanoscale monolithic structure. The contamination of the surface of the separation capillary or channel is due to the non-specific adsorption of analytes present in the sample. The fused-silica capillary or the microchip made of silica material has free silanol groups to which biomolecules such as proteins bind readily. This non-specific binding leads to changes in migration times and peak areas of the analytes after several uses of the separation capillary or channels. In turn, the altered inner surface of the capillary or microchannel yields irreproducible results and limits the use of the capillary or microchip.

To solve these problems, a different approach to introduce samples and buffers was developed, which is known as an “orthogonal” IACE capture-separation system. In this format or protocol, samples, buffers, or solutions are introduced into the ACM in an orthogonal/perpendicular direction via capillaries: in one direction lie the transport capillaries with their entrance and exit ports, while in the other direction lie the separation capillary with its entrance and exit ports. Both the transport and separation capillaries share a small section containing one or more affinity ligands immobilized to a beaded matrix localized within the cavity of the ACM device, or immobilized directly to the inner wall of the capillary or channel. Microvalves are used to control the direction of the passage of fluids, thus preventing contamination of the separation capillary once a sample has been suctioned or pushed through the transport capillary. The transport entrance port is connected to the transport capillary, thereby allowing samples, buffers, or other solutions to be introduced into the ACM device from a container or vessel located at the inlet side of the transport capillary. For this, the microvalves localized at the transport capillary ports are open, and the microvalves localized at the separation capillary ports are closed. The transport exit port allows excess amount of sample or buffer that is passed through to be evacuated into a waste container or vessel. Once a sample has been passed through the transport capillary, allowing for the target biomarker to be captured via immunoaffinity or another affinity principle, a cleaning buffer or solution is introduced through the transport capillary to remove non-specific bound materials. At this stage, the microvalves localized at the transport capillary ports are closed, and the microvalves localized at the separation capillary ports are open. A container or vessel containing a separation buffer or solution located at the inlet side of the separation capillary or channel can then be introduced. The container has a platinum-iridium electrode immersed in the buffer or solution that is connected to a high-voltage power supply, having positive and/or negative polarity. Another container containing the same separation buffer or solution is localized at the outlet end of the separation capillary, having a second electrode that serves to ground the electrical system. A separation buffer is introduced from the inlet to the outlet side of the separation capillary, followed by a small plug of an elution buffer, either alone or carrying a chromophore, to release or release and derivatize the bound target biomarkers from the biorecognition affinity selectors immobilized to the ACM device. A high-voltage power supply, connected to an electrode immersed into the container containing the separation buffer, is then turned on to allow the passage of the target biomarker(s) to the separation capillary exit port. Separation of target biomarker(s) can be accomplished by electrophoretic mobility, electroosmotic flow, mechanical pressure, or a combination of electroosmotic flow and mechanical pressure. The system is hermetically sealed using tight fitted connectors to prevent air bubbles to enter into the capillaries. The separation capillary outlet side can be connected to one or several detectors to quantify and/or characterize the separated biomarker(s). Most detectors are positioned on-line (UV-absorbance or fluorescence), in-line (amperometry), or off-line (mass spectrometry) at the outlet end of the separation capillary, usually at a single point of detection [47]. However, when using a charge-coupled device (CCD) camera detection system, it is possible to capture the sample zones in motion during the migration through the capillary [123].

**Figure 1:**
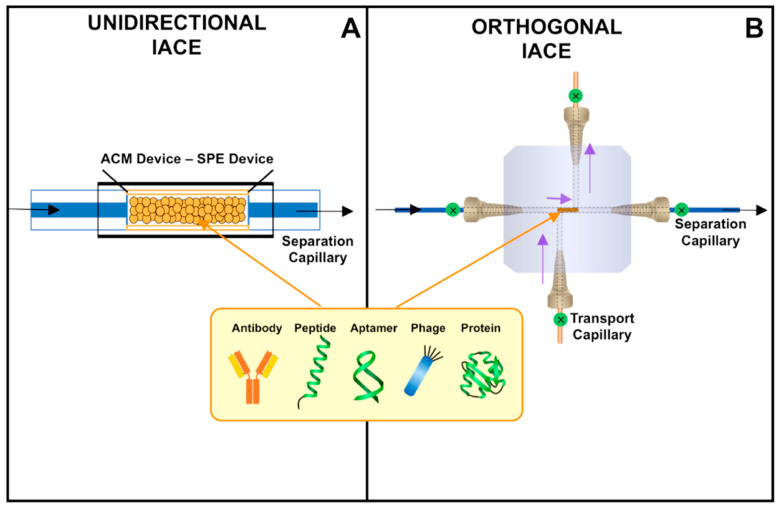
Schematic diagram of two designs of on-line immunoaffinity analyte concentrator-microreactor (IACE) devices. (**A**) Unidirectional IACE design; and (**B**) orthogonal IACE design. The unidirectional IACE design is operated without the presence of microvalves, whereas the orthogonal IACE design requires the presence of microvalves, as depicted in (**B**) with green circles. The orthogonal IACE device has four microvalves positioned at each entrance-exit port. These microvalves are crucial in controlling the path of fluids through the transport capillary or separation capillary. Black arrows indicate flow direction of buffers in the separation capillary, and migration direction of the separated analytes within the separation capillary; purple arrows indicate the flow direction of sample and cleaning buffers introduced into the transport capillary. Biorecognition affinity capture ligands or affinity capture selectors are immobilized to a beaded or monolithic structure positioned within the cavity of the “analyte concentrator-microreactor” (ACM) devices, or immobilized directly to the inner surface of the cavity of the ACM device. The affinity capture selectors can be antibodies, antibody fragments, lectins, aptamers, enzymes, phages, receptors, protein A, protein G, or a variety of substances having affinities for different kind of substances. Figure modified from [25,75].

Figure 1 depicts a schematic representation of the two models of ACM devices. Both containing biorecognition affinity ligands or selectors immobilized to a matrix localized within the cavity of the ACM device. The ACM devices are often referred to as solid-phase extractor devices or simply SPE devices. The term “ACM device” is more appropriate because of the dual functionality of the device: it functions as both an on-line preconcentrator and an on-line microreactor. A major advantage of the orthogonal IACE design is that it permits the protection of the inner surface of the separation of the capillary or channel during the sample introduction and cleaning process. Four micro-valves positioned at each entrance-exit of the ACM device can be controlled manually or by a computer. When the microvalves located at the entrance-exit of the transport capillary are open and the microvalves located at the entrance-exit of the separation capillary are closed, there is no contact of the sample and cleaning buffers with the inner surface of the separation capillary or channel. This arrangement allows for highly sensitive and specific analytical test results.

## 6. Immunoaffinity Capillary Electrophoresis Applications

**Figure 2:**
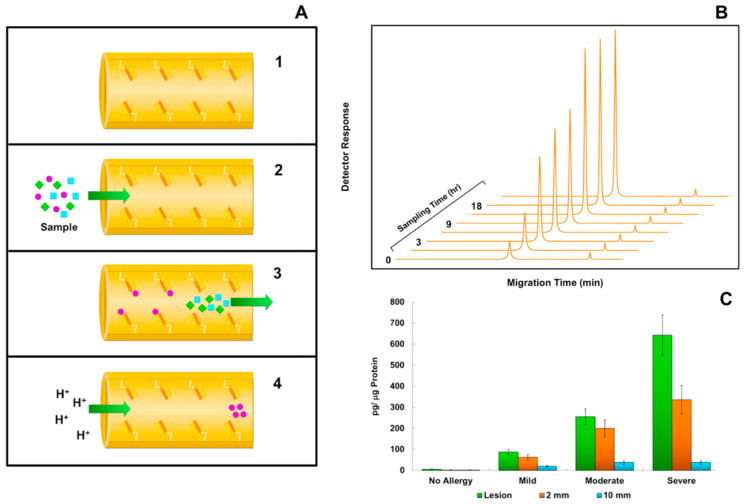
Schematic representation of a fritless ACM device without the presence of a matrix composed of beads, sol-gel, or monolithic materials utilized in conventional capillary electrophoresis or microchip capillary electrophoresis. Immobilization of the affinity ligands occurs directly onto the inner wall of the ACM device (**A**). The protocol for sample processing is shown in four steps: (1) covalent immobilization of an antibody, directed against brain-derived neurotrophic factor (BDNF) neuropeptide used as affinity capture material, directly to the inner wall of a microchip channel (alternatively, 2-mm glass beads immobilized with streptavidin to which a biotinylated antibody against BDNF is coupled, can be inserted into the main channel of the microchip or in an immunoaffinity port of a specially designed microchip); (2) binding of immunoreactive BDNF neuropeptide present in skin biopsies to the immobilized affinity capture antibody; (3) cleaning excess amounts of unwanted non-specifically-bound material and equilibration of the capillary system; (4) elution of the reversibly specifically-bound BDNF neuropeptide. (**B**) Depicts the sequential IACE electropherograms of BDNF concentrations present in skin biopsies taken at different time points in a patient with severe atopic dermatitis illustrating the concentrations of detectable BDNF over time. Graph (**C**) illustrates the concentrations of BDNF in skin biopsy of a patient with severe atopic dermatitis and control groups measured by IACE at 18 h post-initial onset of the reaction. The green color bars represent the amount of BDNF measured within the lesion, while the orange color bars represent the background amounts of BDNF measured in tissues taken 2 mm from the periphery of the lesion. The light blue-colored bars represent the amount of background BDNF measured in tissue taken 10 mm from the periphery of the lesion. All values are the mean ± S.E.M. Figure modified from [55,124].

Numerous immunoaffinity capillary applications employing various configurations of the ACM device, using either a conventional capillary electrophoresis or a microfluidic chip capillary electrophoresis, have been reported [25,54,55,56,57,58,61,75,88,89,90,91,92,93,94,95,96,97,98]. Figure 2 depicts the use of an IACE selective affinity capture-separation system for the quantification of brain-derived neurotrophic factor (BDNF), a neuropeptide growth factor obtained from human skin biopsies of atopic inflammatory reactions [124]. Figure 2A is a schematic representation of an ACM device where whole tetrameric antibodies, nanobodies, antibody fragments, such as Fab, or other affinity ligands, such as aptamers and lectins, can be directly immobilized to its inner surface, instead of using a beaded or monolithic matrix support as depicted in Figure 1. Since the ACM device is not yet commercially available, each laboratory has made its own modifications to the original protocol published elsewhere for use in conventional or microchip capillary electrophoresis [25,66,70,75]. For the protocol carried out in these experiments, streptavidin was first immobilized off-line to the surface of 2-mm glass disks serving as a solid support. This was followed by the addition of biotinylated antibody reacting with BDNF. The prepared disks containing the antibodies to BDNF were then transferred and placed in an area of the microchip termed the immunoaffinity port. Extracted material obtained from frozen tissue biopsies, using a detergent-containing solution, was incubated with the immobilized antibody. The captured BDNF was then conjugated with a fluorescent dye, followed by elution and separation of the derivatized BDNF neuropeptide in the separation channel. Detection was carried out using a laser-induced fluorescence detector. Figure 2B demonstrates that the concentration of BDNF became elevated over a 24-h sampling period in a patient undergoing a severe reaction. Figure 2C shows that the IACE technology is able not only to distinguish differences in the severity of the lesion but could also detect different patterns in analyte concentration at different sampling sites. For example, at the center of the lesion with the highest inflammation area, elevated concentrations of BDNF were found; however, at 2 and 10 mm apart from the lesion, the concentration of BDNF started to decline significantly.

Neuropeptides and their role in regulating inflammatory processes have become a topic of major interest in clinical medicine. BDNF has been associated with several inflammatory conditions, including asthma, hypoxic lung injury, hypersensitivity reactions, atopic dermatitis, neurological disorders, and type 2 diabetes [124,125,126]. In the example presented here for the analysis of BDNF in skin biopsies, IACE technology shows similar results when compared to histopathologic analysis. Furthermore, the example shows that results obtained with IACE can be accomplished in a short period of time, whereas classical histopathology takes several days to complete [124].

**Figure 3:**
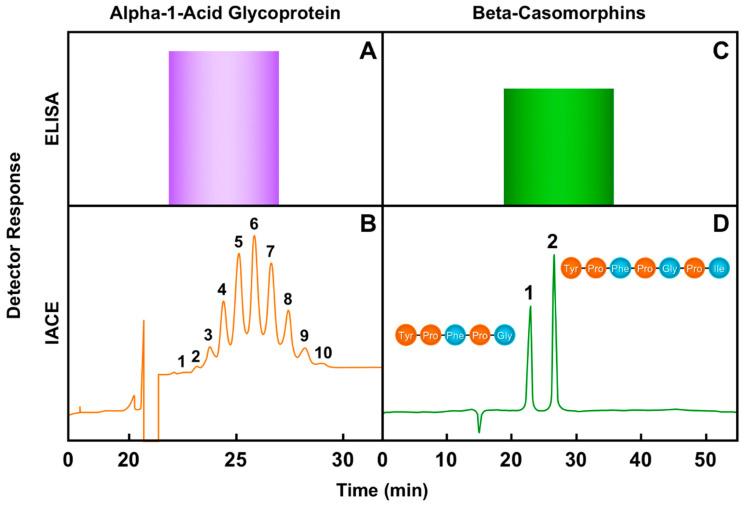
Schematic representation of a comparative profile of enzyme-linked immunosorbent assay (ELISA) and IACE applications for the determination of alpha-1-acid glycoprotein (AGP) or orosomucoid, and for the determination in urine of two structurally similar exorphins, casomorphins 5 and 7. The purple bar (**A**) depicts arbitrary units using an ELISA method for the total cumulative quantitative values of the isoforms of immunoreactive AGP from human serum, and the green bar (**C**) depicts arbitrary units using an ELISA method for the total cumulative quantitative values of the similarly related immunoreactive beta-casomorphin peptides, beta-casomorphin-5 and beta-casomorphin-7, from human urine. Panel (**B**) represents an electropherogram profile for the determination of the isoforms of immunoreactive AGP from human serum, and panel (**D**) represents an electropherogram profile for the determination of the isoforms of immunoreactive beta-casomorphin peptides, beta-casomorphin-5 and beta-casomorphin-7, from human urine. The electropherograms depicted in panels (**B**) and (**D**) were carried out by on-line immunoaffinity capillary electrophoresis. The advantage of determining isoforms of a protein is that each isoform can have various biological roles. Figure modified from [25,127].

Figure 3 depicts a profile demonstrating the data obtained by the two-dimensional IACE immunoassay when compared to the mono-dimensional sandwich enzyme-linked immunosorbent assay (ELISA) method. Two applications are shown utilizing both immunological methods: one is for the determination of alpha-1-acid glycoprotein or orosomucoid, and the other is for the determination of two structurally similar exorphins, casomorphins 5 and 7. The determination of immunoreactive alpha-1-acid glycoprotein (AGP) in serum has been a challenge due to its high carbohydrate content and the heterogeneity in its glycan structures. There are many AGP isoforms or proteoforms of which about 10–20 glycoforms are detected in the serum. The variations of the AGP glycoforms are of interest to the study of the binding alteration of this protein with certain drugs, and of their usefulness as diagnostic biomarkers for some systemic inflammatory diseases [127,128,129]. The quantification of the immunoreactive exorphins derived from milk beta casein A1, casomorphins 5 and 7, is important as these exorphins are potential modulators of numerous regulatory processes in the body as well as potential biomarkers of several diseases. For example, opioid-like casomorphins have been associated with autism, sudden infant death syndrome, type 1 diabetes, apnea, constipation, postpartum psychosis, schizophrenia, circulatory disorders, and food allergies [130,131,132,133,134,135].

As depicted in Figure 3, when the ELISA test is used for the determination of alpha-1-acid glycoprotein and casomorphins, it is unable to detect subtle chemical differences between isoforms of the two protein-peptide molecules. The result is a single measurable detection signal obtained by ELISA, expressed in arbitrary units by a purple bar for alpha-1-acid glycoprotein (Figure 3A), and by a green bar for casomorphins (Figure 3C). Conversely, the results obtained by the IACE technology provide more comprehensive information. Figure 3B shows an electropherogram of the various separated isoforms of immune-reactive alpha-1-acid glycoprotein extracted from serum. Additionally, each peak area of the AGP isoforms can be quantified. The separation of isoforms of particular proteins and their individual quantification can therefore provide significant information for disease diagnosis and prognosis. Clinical proteomic applications aim to solve specific clinical problems by adopting these procedures.

Recent studies are now viewing protein isoforms (proteoforms) as a new class of early diagnostic biomarkers for clinical proteomics [136,137]. Each individual molecular form of an expressed protein has been called a proteoform. This term captures the disparate sources of biological variation that alter primary sequence and composition at the whole-protein level [138,139,140]. The process of identifying aberrantly expressed proteins and disease-associated proteoforms has led to a better understanding of the underlying mechanisms of diseases, particularly tumorigenesis [141]. Figure 3D shows an electropherogram of immunoreactive A1 beta casein-derived casormorphin-5 and casomorphin-7 extracted from urine and analyzed by IACE. Since these peptides have a very similar amino acid composition differing only by one amino acid, ELISA cannot differentiate them given its lack of separation of the peptides after detection. In contract, the two-dimensional IACE technology can separate the two peptides using a single antibody that cross-reacts with both peptides for capture, thereby allowing for their quantification and characterization by one or more detectors. In summary, the mono-dimensional ELISA test is limited to generate a single data point, which provides information only about the sum of the two urine-extracted casomorphins and the 10 serum-extracted isoforms of AGP.

Despite the increase in sensitivity and specificity of immunoassay techniques over the years, analytical interference remains a major area of concern. IACE overcomes many of the limitations of the sandwich ELISA test, in particular the frequent incidence of false-positive and false-negative results for numerous molecules [25,142,143,144,145]. Part of this problem is due to the polyreactive nature of a significant number of antibodies, which bind not only to structurally related targets but also to structurally unrelated targets [146,147,148]. Polyreactive antibodies are a major component of the natural antibody repertoire. In contrast to monoreactive antibodies, polyreactive antibodies have a low binding affinity for antigens, but the antigen-binding pocket of these antibodies is thought to be more flexible than that of the monoreactive antibodies and thereby can accommodate different antigenic configurations [149]. Substances that arise from properties of the specimen and interfere with the reaction of an intended target antigen and reagent antibodies can generate inaccurate results [150,151,152,153]. Consequently, an incorrectly diagnosis that is based on inaccurate testing can lead to inappropriate and, in some cases, harmful treatment.

In addition to false positive results, ELISA can lead to false negative results. This tends to arise from weak binding affinity of the antigen to the affinity-capture agent. A number of modifications have been recommended to avoid interferences with antigen binding in immunoassays, such as the addition of detergents, treatment of a sample at high temperatures if the analyte is thermal stable, addition of some blocking agents to a sample, and others. However, despite advances in our knowledge and understanding of the mechanisms of interference in immunoassays, there is no single procedure that can rule out all interferences with ELISA or other immunoassays [150]. The development of a portable point-of-care IACE biomarker analyzer instrument that utilizes two-dimensional IACE technology offers a potential solution to improve the conditions of binding between the affinity-capture molecule immobilized to the ACM device and the target analyte to be tested. For example, addition of small amounts a non-ionic detergent, such as of Nonidet P-40, addition of certain polycations, such as polybrene, and maintenance of the optimal ionic strength of the buffer, can help to improve the binding between the target analyte and the affinity-capture biorecognition molecule. Other factors are also crucial to optimize binding, such as the control of temperature, buffer pH, and time of reaction; the use of a microwave pulse or acoustic micro-mixing system; and minor sample dilution with an appropriate conditioning buffer, all of which can improve binding and reproducibility of the assay [25,75,120,121,122,154,155].

Another major advantage of the design of the IACE biomarker analyzer instrument is that if the biorecognition affinity ligands or selectors (e.g., antibody, antibody fragment, lectin, aptamer, others) are stable and covalently immobilized with a well-defined orientation, then they can be reutilized multiple times. The advantage of this optimization protocol is that it permits the use of multiple well-oriented affinity-capture agents with high affinity and selectivity, in order to secure the binding of the target analyte. The ability of the ACM device to be reused therefore brings down the cost per assay, resulting in significant benefits to patients that need frequent analysis of biomarkers.

**Figure 4:**
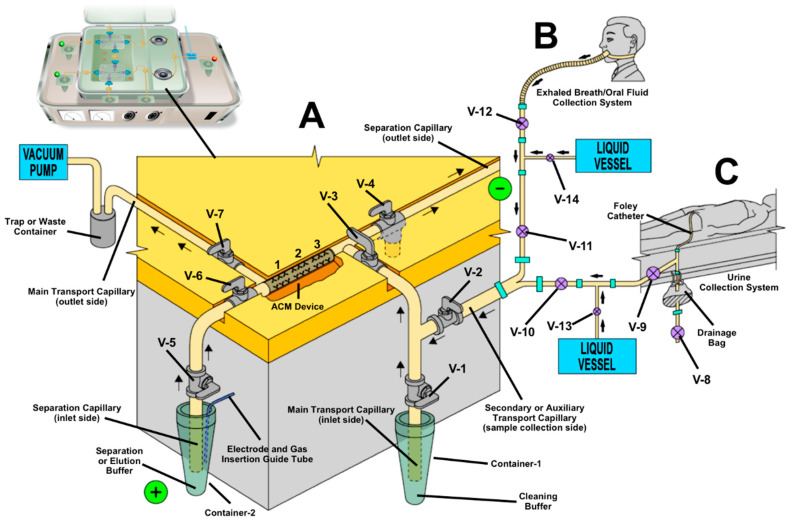
Schematic representation of a modified version of a portable point-of-care IACE biomarker analyzer instrument (**A**) coupled to two biological specimen collection systems; an exhaled breath/oral fluid collection system (**B**), and to a urinary collection system composed of a urinary drainage bag connected to a Foley catheter (**C**). The two sample collection systems can work in synchronization with the point-of-care IACE biomarker analyzer instrument in a separate and sequential order using control microvalves (V-1 to V-14) to regulate the flow of collected samples from an individual. The ACM device having a staggered configuration, can have one or more biorecognition affinity ligands immobilized to its inner wall to capture one or more similar or distinct biomarkers present in the collected samples. The point-of-care IACE biomarker analyzer instrument can also be equipped with one or more ACM devices (depicted in the upper left side), all having a staggered configuration, with the purpose to capture, concentrate, separate, and analyze a panel of biomarkers. By generating a comprehensive set of biomarker information from the biological specimen, the instrument aims to obtain a reliable and accurate diagnosis, prognosis, and to provide an efficient method for the assessment of treatment efficacy. In addition to capturing and analyzing chemical and biochemical compounds, the ACM device of the portable point-of-care IACE biomarker analyzer instrument is capable of capturing circulating blood cells, subcellular entities, cell debris, viruses, exosomes, and the components of disrupted biological particles. To facilitate a smooth passage of the fluid through the tubes or capillaries, a detergent-containing solution, stored in the liquid vessel or container, is added to the system during the collection of the sample. Moreover, a few filters are incorporated into the tubes or capillaries, containing digestive enzymes immobilized to their constituent matrices, with the purpose of breaking down larger biomolecules, cell aggregates, cell debris, or any material that may block the filters and may disturb the smooth flow of the biofluid throughout the transport tubes or capillaries. The integrated IACE biomarker analyzer instrument can be used in remote areas, in ambulances, and in intensive care units of a medical facilities, and it can be installed under a protective and confined space for patients with infectious diseases. The data collected can be sent by a secure and codified system using Web portals via Internet to a supercomputer to reveal comprehensive biomarker diagnostic signatures, thereby providing accurate and effective diagnosis information to a healthcare professional, and back to the computer of the IACE biomarker analyzer instrument to keep the information updated and stored. Figure modified from [75,122,155].

Figure 4 shows a schematic representation of a modified version of a portable point-of-care IACE biomarker analyzer instrument (Figure 4A) coupled to two biological specimen collection systems: an exhaled breath/oral fluid collection system (Figure 4B), and a urinary collection system composed of a urinary drainage bag connected to a Foley catheter (Figure 4C). The two sample collection systems can work separately and in synchronization with the point-of-care IACE biomarker analyzer instrument in a sequential order.

The ACM device depicted in Figure 4A is structurally positioned in order to prevent the contact of the sample with the separation capillary [25,75,120,121,122,154,155]. This task is performed with the help of four microvalves surrounding the ACM device. A biological specimen containing one or more target analytes to be tested is obtained either by the exhaled breath/oral fluid collection system Figure 4B or by the Foley catheter urine collection system Figure 4C. The corresponding specimen is then transported through a secondary or auxiliary transport capillary to the main transport passage or capillary, passing through the ACM device containing three biorecognition affinity ligands (1, 2, and 3) all the way to the outlet end of the main transport capillary to a trap or waste container.

Sample introduction is carried out from the outlet side of the secondary transport passage or capillary, connected to the main transport passage or capillary, by controlled suction using a vacuum pump coupled to a trap or waste container collecting the excess amount of fluid. Microvalves located at the inlet side of the main transport capillary (V-1) and at the inlet and outlet side of the ACM device, which is positioned at the separation capillary (V-4 and V-6), are closed. Microvalves located at the inlet and outlet side of the ACM device, positioned at main transport capillary (V-3 and V-7), are open. When collecting exhaled breath/oral fluid, the microvalves positioned at the exhaled breath/oral fluid collection system (V-11 and V-12) are open, and the microvalves positioned at the Foley catheter collection system (V-8, V-9, and V-10) are closed. The microvalve (V-2), positioned at the secondary or auxiliary transport capillary is open. When collecting urine, the microvalves positioned at the Foley catheter urine collection system (V-9 and V-10) are open, and the microvalves positioned at the exhaled breath/oral collection system (V-11 and V-12) are closed. Microvalves V-13 and V-14 are usually open, allowing a detergent-containing buffer to mix with the exhaled breath/oral fluid or urine sample to facilitate solubility and avoid aggregation of molecules. This maintains smooth flow of the collected samples through the various passages or capillaries of the transport system, from the collection area to the outlet of the main transport capillary passing through the ACM device.

After binding occurs between the target analyte(s) present in the biological specimen and the biorecognition affinity ligands immobilized to the ACM device, the microvalve (V-2), positioned at the secondary or auxiliary transport capillary is closed, and the microvalve positioned at the inlet side of the main transport capillary (V-1) is open. A cleaning buffer or solution is introduced through the container positioned at the inlet side of the main transport capillary (Container-1) to remove non-specific compounds bound to the inner surface of the main transport capillary.

Once the binding and cleaning processes are completed, microvalves positioned at the inlet and outlet sides of the transport capillary (V-3 and V-7) are closed, and the microvalves positioned at the inlet and outlet sides of the separation capillary (V-4, V-5, and V-6) are open. A separation buffer is introduced into the separation capillary from a container positioned at the inlet side of separation capillary (Container-2) to a container localized at the outlet side of the separation capillary (not shown). A plug of an elution buffer can be introduced into the inlet side of the separation capillary by pressure, using an inert gas such as argon or helium, followed by the separation buffer. Electroosmotic flow can also be used for the introduction of a plug of an elution buffer. When the high-voltage power supply connected to a platinum-iridium electrode is switched on the process of elution and separation starts. At the outlet side of the separation capillary, eluted and separated target analytes are detected, quantified, and partially characterized by one or more detectors connected to the capillary in-line (amperometry), on-line (UV-absorbance or fluorescence), or off-line (mass spectrometry).

As illustrated in Figure 4A, the ACM device has three types of affinity ligands (1, 2, and 3) immobilized to its inner surface representing three different affinity-capture molecules. The biorecognition affinity ligands can be an antibody, a lectin, and an aptamer, or a combination of the three (i.e., three different antibodies). Similarly, the target analytes can be three different target analytes or a single target analyte that has three different epitopes.

With the advent of advanced technologies offering powerful bioengineering tools, it is possible to create new affinity-recognition-capture molecules with improved and unique binding capabilities. For example, nanobodies, or single-domain variable fragments of camelid-heavy chain-only antibodies, have been successfully engineered as highly selective reagents, with high affinity, minimal size, low cost, and great stability. Nanobodies are used as research tools and in medicine [156,157,158]. Similarly, aptamers, which are short, single-stranded oligonucleotides that bind to specific target molecules, are playing an important role as affinity recognition-capture and therapeutic reagents [159,160,161]. Another important group of affinity-recognition-capture reagents are lectins, which comprise a widespread group of sugar-binding proteins occurring in all types of organisms including animals, plants, bacteria, fungi, and even viruses. They are used as an effective tool for the targeting, separation, and reliable identification of glycoprotein molecules. Their importance stems from the understanding that changes in glycoprotein and glycopeptide content, altered glycosylations, and aberrant glycan structures are increasingly recognized as cancer hallmarks [162,163,164,165].

The use of two or more affinity-recognition-capture reagents has been demonstrated to improve the affinity binding to a molecule with multiple epitopes, such as thrombin [166]. Two different oligomeric DNA aptamers that can recognize different epitopes in thrombin were used in a potentiometric biosensor. When testing for the detection of low concentrations of thrombin, the dual aptamer-immobilized configuration yielded better results than the one with a single aptamer when tested for the detection of tiny amounts of thrombin [166]. Another application employing multiple affinity-capture agents has been reported to use six different antibodies to capture six different immunoreactive chemokines in samples of cerebral spinal fluid collected from premature babies. In such study, immunoaffinity capillary electrophoresis in microchips was used to determine the degree of brain trauma in birth traumatized premature babies. The quantification of six different chemokines was able to provide information for diagnosis and prognosis of the good and poor state of the babies with head trauma [167]. Additional examples of using multiple antibodies to capture several biomarkers have been presented in [25,55,58,155].

The main advantage of the ACM device, having multiple affinity-recognition-capture reagents (as depicted in Figure 4A) is its capability to analyze a panel of biomarkers simultaneously in a single platform to generate a comprehensive data and information. For example, it is possible to obtain the following information from a single biosample analysis from a patient suffering from a chronic or an infectious disease or illness and undergoing treatment: (1) the presence of the antigen causing the disease, such as a microorganism or its components of the microorganism if the microorganism is broken apart; (2) the presence of one or more antibodies, if the patient’s immunological system recognizes the microorganism or its components as foreign substances consequently responds by developing antibodies to the unknown antigens; (3) the presence of pro-inflammatory substances and anti-inflammatory substances, if the patient’s body is fighting the disease as it develops; and (4) the presence of other molecular markers expressed by the body or by the cellular or subcellular entities, and secreted to the biological specimen during the development of the disease. In the case of a comprehensive collection of information from the isolation and quantification of a panel of biomarkers in the biosample(s), it should be feasible to make an accurate and reliable diagnosis of the disease, a prognosis of the disease (i.e., if it will continue its progress, or if the patient has hope for recovery), and a pathway for monitoring the effectiveness of treatment (if any). Furthermore, because of the high power of resolution of capillary electrophoresis, it is achievable to isolate and characterize novel co-translational and/or post-translational modifications of biomolecules, whether during the development of the severity of the disease or as a result of other biological parameters that may serve as better and more promising biomarkers of predicting clinical outcomes.

The accuracy, analytical sensitivity, and reproducibility of the tests obtained by the two-dimensional IACE technology are higher than those of conventional mono-dimensional immunoassays such as ELISA. Most notably, there is little to no possibility of false-negative or false-positive results when using IACE, whereas such results can be obtained with some frequency when using ELISA. For example, if a mono-dimensional ELISA test captures unrelated immunoreactive compounds, then a false-positive result may be obtained due to the polyreactivity of antibodies which are capable of interacting with related and unrelated substances. On the other hand, if a similar situation happens with the two-dimensional IACE technology (wherein the test captures related and unrelated immunoreactive compounds), there still exists the possibility of confirming the identity of each separated peak using additional tools and detectors capable of providing partial or complete characterization of the separated analytes (e.g., by spiking the sample with internal standards, determining the corresponding migration time of each peak, and obtaining the absorption spectral profile and mass spectrometry profile for each separated compound).

An enhancement to the features of the portable point-of-care IACE biomarker analyzer instrument as described in Figure 4A can be accomplished by coupling the instrument, whether as an integral component or as a detachable unit, to a portable-point-of-care breath/oral fluid collection system as depicted in Figure 4B and described elsewhere [122,168]. Breath analyzer systems have been reported for the determination of organic volatile compounds and ionic content in exhaled breath condensate [169,170]. However, studies of exhaled breath have demonstrated that humans generate fine particles during tidal breathing. Micron and submicron particle sizes have been detected in exhaled breath of normal and pathological people [171]. Exhaled breath sampling and analysis has long attracted interest in the areas of medical diagnosis and disease monitoring [171,172,173,174,175,176,177]; however, progress from laboratory settings to routine practice has been slow. One main reason is the number of technical problems encountered for sampling and analysis, and the lack of normalization and standardization leading to significant variations that exist between results of different studies [174].

Mouth-exhaled breath contains not only volatile organic compounds, but many other small and large molecules, as well as cellular and subcellular particles. These biomolecular and cellular entities originate from the airway, from the oral cavity and gut by bacterial action, and from mucus and saliva. Among the substances found in exhaled breath are inflammatory cytokines. It is well-known that inflammation is part of the host’s protective immune response against microorganisms; on the other hand, excessive inflammation can be detrimental to patients’ health as it increases their morbidity and mortality. For example, dysregulation between pro- and anti-inflammatory cytokines has been found in exhaled breath obtained from patients with community-acquired pneumonia [178].

The potential of the portable point-of-care IACE biomarker analyzer instrument to serve as a dual-integrated and complementary unit is greater than that of traditional instrumentation, since the instrument becomes a multi-modal screening system capable of isolating, concentrating, and quantifying multiple biomarkers for a comprehensive diagnostic and prognostic test. A panel of biomarkers has the potential to detect cases missed by the use of only a few biomarkers. Infectious diseases, whether bacterial, viral, or of other origins, present acute and chronic stages of inflammation. Early detection remains the key challenge to the survival of patients with contagious infectious diseases that may spread rapidly from one organ to another and can cast a storm over the whole body, ending in multiple organ failure. A cytokine storm syndrome having an hyperinflammation and characterized by elevated levels of cytokines in biological fluids can be a predictor of fatality. In particular, increased levels of interleukin-2, interleukin-7, granulocyte-colony stimulating factor, interferon-gamma inducible protein 10, monocyte chemo-attractant protein 1, macrophage inflammatory protein 1-alpha, and tumor necrosis factor-alpha have been associated to mortality due to virally driven hyperinflammation, as for example, in coronavirus disease 2019 (COVID-19) [179].

Figure 4C depicts an additional modular attachment to the portable point-of-care IACE biomarker analyzer, with a different configuration from the one described in Figure 4B. In this case, sample collection is performed from a connecting insertion to a modified urinary catheter, also called a Foley catheter, as used in a catheterized patient. Urinary catheters are used to help to empty the bladder, usually when a person is unable to urinate. The catheter is a sterile and flexible tube that is inserted through the urethra into the bladder. Often urinary catheters are used during surgery, as the patient is unable to control their ability to urinate during anesthesia. In most cases, the catheter is intended to stay in place for a long period of time. Urine drains from the bladder through the tube and into a collection bag. In addition to urinary retention and during surgery, the catheter is also used in many patients who stay in a hospital’s intense care unit (ICU) and who are too sick to use a bedpan. Unfortunately, some complications such as urinary tract infections, may arise when catheters are used for an extended period of time [180]. In most cases, urinary tract infections are commonly treated with empirical antibiotics, resulting in overuse of antibiotics, which promotes antimicrobial resistance. Interestingly, when urine from patients suffering from urinary tract infection is cultured, approximately only one in three patients are found to have urinary tract infection as defined by positive bacterial culture [181]. Results from bacterial culture may take from one to three or more days, depending on the type of bacteria [182].

The portable-point-of-care IACE biomarker analyzer instrument can overcome this delay since it is capable of generating comprehensive information based on a panel of biomarkers analyzed in the urine collected from the modified Foley catheter collection system. The panel of biomarkers may include small molecules, biomolecules, and cellular and subcellular particles. These particles can be microorganisms such as bacteria, viruses, and others, including exosomes which are considered to be a trove of biomarkers [183]. A panel of biomarkers should therefore be able to confirm the presence or absence or a microbial infection. It has been reported that isolation and characterization of a panel of host-protein biomarkers yields significantly superior performance for diagnosing bacterial versus viral infections [184].

There is always uncertainty on how to manage a patient who may be a potential carrier of a contagious disease, as evident in the recent coronavirus pandemic. A “patient under investigation” is often placed in conservative precautions, and healthcare teams may defer or avoid certain procedures which may have been otherwise performed in other patients [185,186]. To provide physicians with the answers they need to manage patients effectively during an outbreak setting, laboratory testing is needed on the front lines whenever it is feasible and safe to do so. In Figure 4 we describe a portable-point-of-care IACE biomarker analyzer that can be placed near the bedside of a patient and be separated by a plexiglass wall, such as the biomarker analyzer instrument that can be operated from an isolated containment area if necessary. When used on a patient, the portable and modular breath/oral collection system placed on a patient can be connected to the main instrument using a long flexible tube through the plexiglass wall to avoid any contamination. A similar connection can be performed when using the modified Foley tubing coupled to the main instrument. The main transport and secondary or auxiliary passages or capillaries have an internal diameter usually between 500 and 900 µm, permitting enough surface area for passing fluid derived from the exhaled breath and urine specimens and their respective contents. The Foley tubing and the exhaled breath/oral collection system, respectively, have much larger internal diameters. The average size of most bacteria is between 0.2 and 2.0 micrometers, but others may be as large as 10 micrometers. White and red blood cells have size ranges fluctuating between 6.0 and 18 micrometers on average, and viruses, nanobacteria, or extracellular vesicles (EVs) have size ranges primarily in the nanometer scale. The separation capillary used in conventional capillary electrophoresis instruments usually ranges between 50 and 100 micrometers, even though other internal diameters smaller than 50 micrometers or larger than 100 micrometers can be used as well. Therefore, for most cases it should not be a problem to isolate and characterize cellular, subcellular, and extracellular particles when using the portable-point-of-care IACE biomarker analyzer instrument.

As illustrated in Figure 4A–C, the interchangeable modular infrastructures designed for the rapid analysis of a comprehensive biomarker information, including a plexiglass wall for keeping the entire functional instrument in containment, should enable a successful robust and sustainable system which protects the patient as well as the healthcare workers. All technical operation of the biomarker analyzer instrument (devices, attachments, connectors, and valves) can be performed remotely via computer using robotic manipulators or controllers, and thus without the risk of any physical contact. Robotic manipulators are capable of performing repetitive tasks at speeds and accuracies that far exceed those of human operators. Modifications to the connection systems and other parts of the instrument and devices, including change of buffers and solutions, can be carried out as needed in a simple manner. The modularity of the components of the instrument and devices allows for the rapid replacement of parts. Additionally, attachments of extra miniaturized detectors for improving sensitivity and characterization should facilitate significantly the detection and characterization of substances and/or particulate matters found at very low concentrations.

## 7. The Potential Use of IACE for Extracellular Vesicle Studies

Early medical practitioners realized the importance of counting blood cells as a tool for investigation and quantitative study in healthcare [187]. For over 150 years, cell biologists had been using a hemocytometer (counting chambers) to quantify cells (mm). Today, automated instruments such as flow cytometers are widely used in research and for management of patient diagnosis and prognosis, particularly for blood cancer cells [187,188]. Significant effort has been made to analyze cellular and subcellular entities, as well as viral particles and cellular vesicles using a flow cytometer (recently dubbed “flow virometry”) [189], but the straightforward applicability of this technique has been hampered by the small sizes of the particles and vesicles, their polydispersity, and the low refractive index [190,191,192].

With the rise of microfluidic devices, several attempts have been made to develop cell counters using microchips. However, for microfluidic devices to supplant current cytometers, it will require more development in sample preparation and sample techniques within lab-on-a-chip microfluidic platforms or miniaturized capillary electrophoresis instruments. Studies on single cellular and subcellular entities, as well as their cargo content, by using conventional capillary electrophoresis and microchip capillary electrophoresis have been reported [193,194,195,196]. There are several benefits associated with using these platforms, including low-volume requirements, rapid and efficient separations, special dimensions, and versatility. However, for the isolation and separation of a heterogeneous group of cellular and subcellular entities, as well as viral particles and cellular vesicles, many parameters must be taken in consideration, and the challenge for technical improvements will continue for a few more years.

**Figure 5:**
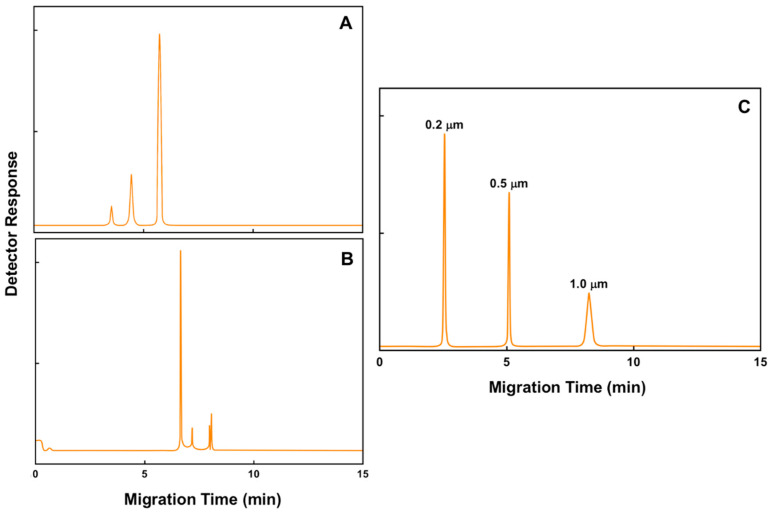
Schematic representation of capillary electrophoresis separation of two types of biological-like particles and one polymeric particle. (**A**) Electropherogram of human rhinovirus serotype 2 (main peak corresponds to the human rhinovirus serotype 2 virus (HRV); smaller peaks may be contaminants of the preparation). (**B**) Electropherogram of *Pseudomonas fluorescens*, a Gram-negative, rod-shaped bacterium (main peak corresponds to the bacterium; smaller peaks may be contaminants of the preparation). (**C**) Electropherogram of polystyrene particles based on their sizes. Figures modified from [205,206,207].

We are proposing a platform, based on IACE, as a complementary tool to the existing technologies for the isolation and separation of particles of small sizes, such as viruses and EVs. As demonstrated in Figure 5, capillary electrophoresis has already been used with some success for the separation of bacteria, viruses, and polymeric particles. It would be beneficial to have a sample processing method before separation, to isolate and concentrate the intended viruses or EVs. Immunoaffinity capillary electrophoresis has already been proven to be a useful technology to isolate, separate, and quantify cell-free molecules of biological interest based on the specificity and selectivity not only of antibody reagents, but also of lectin and aptamer reagents, quantifying molecules ranging from microgram/milliliter to femtogram/milliliter [25,54,55,57,75].

There are several reasons why we are interested in using the IACE technology to study viruses and EVs, in particular exosomes. The first reason is because we are in the middle of a pandemic, and the study of SARS-CoV-2 has emerged as a priority subject of research for the medical and scientific community [179]. The second reason to apply IACE to study exosomes is because of the potential of these nanoparticles in clinical diagnosis and prognosis, as well as carriers of therapeutic drugs [197,198,199,200,201,202,203,204]. Not only are exosomes released by basically all cell types, they are also accessible in body fluids, they carry important molecular compounds of potential use as biomarkers, and they can cross many cellular barriers. As such, they play an important role in many physiological processes and have been implicated in many diseases [197,198,200]. As EVs encompass a heterogeneous group of cell-derived vesicles, it would be a challenge to isolate, concentrate, and separate the three groups of nanoparticle entities.

In view of the fact that there is a significant interest in the study of viruses and exosomes, a brief discussion of their importance is warranted. Extracellular vesicles are a heterogeneous group of cell-derived membranous structures which originate from the endosomal system or which are shed from the plasma membrane, respectively. Extracellular vesicles have been classified as oncosomes (1–10 micrometers), apoptotic bodies (500–2000 nanometers), microvesicles (50–1000 nanometers), and exosomes (40–200 nanometers) [197,198]. Some even smaller vesicles known as “exomeres” have been described as having even smaller sizes [199]. Exosomes are naturally occurring nanosized vesicles and consist of natural lipid bilayers with an abundance of adhesive proteins that readily interact with cellular membranes. These vesicles’ contents include cytokines and growth factors, signaling lipids, mRNAs, and regulatory miRNAs. Exosomes have many roles and functions, and they are found in body fluids, including plasma, serum, urine, saliva, exhaled breath condensate, breast milk, tears, semen, cerebrospinal fluids, amniotic fluid, malignant ascites fluids, and cultured medium of cell cultures [197,198,199,200,201,202,203,204]. They are involved in multiple physiological and pathological processes [197,198,199,200,201,202,203,204]. These extracellular vesicles represent an important mode of intercellular communication by serving as functional vehicles that carry a complex cargo of proteins, lipids, and nucleic acids, and altering cell or tissue metabolism, influencing tissue responses to injury, infection, and disease [200,201,202,203,204]. Exosomes are heterogeneous in size, heterogeneous in composition, and enriched in membrane-associated, high-order oligomeric protein complexes.

Exosomes and many types of enveloped viral particles, particularly RNA viruses, have a similar size making exosomes and RNA viruses “close relatives” [202]. In fact, enveloped viruses may be considered a form of EV. Exosomes from virus-infected cells incorporate not only cell-encoded but also virus-encoded molecules [202]. Exosomes are nanoparticles in the range of 40–200 nm (average 100 nm). Therefore, they can be separated by capillary electrophoresis as many other biological particles have been separated. For example, Figure 5 shows the separation by capillary electrophoresis of a virus (human rhinovirus serotype 2) [205], a bacterium (*Pseudomonas fluorescens*, a Gram-negative, rod-shaped bacterium) [206], and three polymeric particles (polystyrene micron-sized particles) [207]. The highly selective affinity capture of biorecognition agents such as antibodies, lectins, and aptamers, in conjunction with the superior resolving power of capillary electrophoresis, make a perfect pair to concentrate and separate biological species or entities for potential use in diagnosis. A typical example are viruses, which usually exist as more than one type. Dengue infections are caused by four closely related viruses named DEN-1, DEN-2, DEN-3, and DEN-4. These viruses are called serotypes, because each type of virus has different interactions with the antibodies in human blood serum. They are mosquito-borne flaviviruses responsible for dengue fever and severe dengue hemorrhagic fever that belong to the genus Flavivirus, family Flaviviridae, which contains approximately 53 viruses. The flaviviruses are relatively small (40–65 nm) and spherical with a lipid-rich envelope [208]. Vaccination must protect against all four serotypes, and hence this has proved to be difficult to design. Structural differences between dengue viruses produced in humans versus cell lines many be key to understanding vaccine failure and developing better models for vaccine evaluation [209]. In the case of the coronaviruses, there are seven coronaviruses known to infect humans, but only three of them, SARS-CoV, MERS-CoV, and SARS-CoV-2 cause severe disease in humans. The seven coronaviruses are (1) 229E (alpha coronavirus), (2) NL63 (alpha coronavirus), (3) OC43 (beta coronavirus), (4) HKU1 (beta coronavirus), (5) MERS-CoV (the beta coronavirus that causes Middle East Respiratory Syndrome, or MERS), (6) SARS-CoV (the beta coronavirus that causes severe acute respiratory syndrome, or SARS), and (7) SARS-CoV-2 (the novel coronavirus that causes coronavirus disease 2019, or COVID-19) [210].

Little is known about the coronavirus SARS-CoV-2, which is responsible for the novel pneumonia known as coronavirus disease 2019 or COVID-19. It appears that day after day, physicians and scientists are learning new information about the transmissibility and severity of the epidemic it causes. The seriousness of these infections and the lack of effective, licensed treatments for SARS-CoV-2 infections underpin the need for a more detailed and comprehensive understanding of coronaviral molecular biology, with a specific focus on both coronaviruses’ structural proteins as well as their accessory proteins [211]. The known vaccines typically work by eliciting antibodies that block entry of the pathogen into cells, which in the case of enveloped viruses, involves antibody binding to the viral envelope proteins. The viral fusion protein is the key factor that induces the membrane fusion reaction that in turn allows viral entry [212]. Analyzing structural biology of the viral membrane fusion proteins and their complexes with neutralizing antibodies is thus an exceptionally powerful approach to identifying vulnerability sites and to extracting the necessary information required to develop protective vaccines to efficiently combat the emerging viral diseases threatening our planet [212]. Immunoaffinity capillary electrophoresis can serve as a platform to isolate, concentrate, and analyze various structurally related viruses, and to analyze their chemical modifications they might undergo, thereby providing significant information about a diagnostic signature classification of their serotypes. Similarly, this approach can be implemented to study extravesicular vesicles and various types of circulating cells, such as immune cells and cancer cells [213,214], as well as quantifying and analyzing their chemical and biochemical contents.

Capillary electrophoresis can separate molecules or entities based on the differences in the charge-mass ratio of single molecules, complexes of molecules, circulating cells, biological particles, and even man-made nanoscale quantum dots [215]. Recently, it has been proposed as a proof of concept that capillary electrophoresis can be used for the separation of bacterial extracellular vesicles [216]. Much effort has been performed during the last 5 years to develop methods for the isolation and characterization of exosomes, which are seen as attractive candidates for clinical application as innovative diagnostic and therapeutic tools [217,218,219,220,221,222,223,224]. Lately, there has been increasing evidence which demonstrates that the positive effects of cell-based therapies are mediated by exosomes released from the administered cells, and that the micro-RNA cargo in these exosomes is largely responsible for the therapeutic effects [225]. Advances in the study of exosomes have been delayed because of the lack of methods to isolate them from clinical samples. Traditionally, differential ultracentrifugation, ultrafiltration, immunoaffinity capture, and size exclusion chromatography have been used as conventional methods of isolation, but these methods are generally time-consuming and labor-intensive. They also may need costly instrumentation or some special chemical reagents, and in some cases, such methods of isolation may require multiple overnight centrifugation steps [226,227].

In the last few years, several characterization and validation methods have been developed, for both research and clinical purposes, to analyze exosome purity and to quantify exosomal cargo. These methods include transmission electron microscopy (TEM), scanning electron microscopy (SEM), atomic force microscopy (AFM), nanoparticle tracking analysis (NTA), dynamic light scattering (DLS), resistive pulse sensing, enzyme-linked immunosorbent assay (ELISA), flow cytometry, fluorescence-activated cell sorting (FACS), and microfluidics and electrochemical biosensors [228,229,230,231,232]. The number of methods for quantifying exosomes has expanded as interest in exosomes has increased. However, consensus on proper quantification has not developed, making each study difficult to compare to another [233]. Immunologically, exosomes exhibit antigen-presenting capability [234]. As a result, immunoaffinity studies of exosomes are becoming attractive, and IACE can be an ideal platform to be used for such applications in research and in clinical diagnosis. In fact, a few laboratories are already combining immunoaffinity and microfluidic system approaches for more efficient exosome collection [235,236,237,238,239].

Given their biological activities in intercellular transportation, information communication, and cell-mediated immunity modulation after microbial infection, exosomes are considered to be of significant value for a comprehensive understanding of infectious diseases as they play a key role in clinical defense of microbial infections. Furthermore, exosomes will play a significant role as immune drug carriers, both in the manufacture of biological vaccines, and as biomarkers of microbial infections [240]. Recently, it has been shown that exosomes serve as decoys, providing cellular protection against bacterially produced toxins [241]. A consistent high-quality isolation method for exosomes, followed by characterization and identification of types exosomes and their content, is therefore crucial to distinguishing subpopulations of exosomes, other extracellular vesicles, and viral particles. A conceptual understanding of biological responses to nanoparticles is likewise needed to develop and apply safe nanoparticles in drug delivery and other uses [242].

The future of the in-depth investigation of exosomes will rely heavily on technological advances, particularly with respect to simplifying the capture of functional microvesicles, such as exosomes, and to providing accurate quantitative measurement of their contents [243,244]. Many interesting findings and hopes for advancing diagnosis and therapy for exosomes have been reported recently (Table 1). One such study has found that exosomes released in response to cigarette smoke might trigger chronic obstructive pulmonary disease (COPD), whereas engineered versions could be used as treatment of the disease [243,244,245]. Another report has shown that exosomes from virus-infected cells modulate immune cells’ responses and increase spread and infection of the virus through delivering viral nucleic acid and protein molecules to healthy cells [246].

As discussed earlier in this paper, the two-dimensional IACE technology provides many advantages when compared to other immunoassays for the analysis of cell-free molecules, such as the use of low volumes and reagents, high analytical sensitivity, speed of analysis, and the separation of all target and non-target affinity-captured analytes [25,54,55]. Nevertheless, there are a few technical challenges for the application of this technology to the isolation, concentration, and separation of cellular particles and vesicles which remains unresolved [247,248]. The goal of analyzing exosomes by IACE is to further study the association of the cargo content of exosomes with the presence or absence of a wide range of pathogens, such as bacteria or viruses, and integrates the host injury response to infection [13]. Moreover, by primarily quantifying nucleic acids and crucial protein biomarkers which serve as indicators of early detection and evolution of diseases, exosomal analysis employing IACE can further provide information about the injury of different tissues in a patient due to pathogens and/or inflammatory substances. The role of non-coding RNAs (ncRNAs) in EVs is not fully understood, but their higher abundance in association with cancer and other diseases demonstrated their relevance. Several studies are underway on microRNAs, long ncRNAs, and circular RNA present in exosomes, and evidence exists that non-protein coding genes harbors crucial importance in terms of development, homeostasis, and disease [249,250,251,252,253,254].

## 8. Conclusions

The reliability of biomarkers in day-to-day medical practice still appears to be in question because of a lack of access to extremely sensitive and specific diagnostic testing. Researchers are therefore not only discovering more comprehensive ways to determine disease activity via biomarker detection, but also linking biomarkers to risk factors that would allow for better prediction models to be created for risk of disease manifestation [5]. Clinical implementation of exosome-based diagnostic and therapeutic applications is still limited by the lack of standardized technologies that integrate efficient isolation of exosomes with comprehensive detection of relevant biomarkers. With the IACE biomarker analyzer instrument and its interconnected sampling collection systems as described in this manuscript, we expect to accurately identify and quantify different morphological subpopulations of nanovesicles and their components in a single platform. We hope that this device can be affordable and readily available in a clinical setting in the near future once biomarker applications have demonstrated its clinical validity, and once analytical validation studies have demonstrated its accuracy, precision, reproducibility, and sensitivity levels. However, as in all great technological revolutions, IACE has and may continue to have some limitations. The advantages of this two-dimensional affinity capture-separation technology have just begun to come into view over the past few years, and the trend in usage of IACE in clinical contexts is moving consistently in the positive direction. Most promisingly, the incorporation of IACE into telehealth, with the intent to meet the needs of telemedicine, and precision medicine, is beginning to revolutionize the delivery of healthcare to all segments of the population. In the near future, we foresee that the use of the IACE technology, in conjunction with liquid biopsy, for the study of circulating cell-free molecules, single cells, subcellular entities, viruses, exosomes, and their constituent components in biological fluids, will become a powerful tool to unravel longstanding questions in both biological research and clinical diagnostics.

## Figures and Tables

**Table 1:** A number of publications related to the potential use of exosomes obtained from liquid biopsy or cultured cells, and their cargo contents in diagnosis, prognosis, and therapy.

Source	Clinical Significance	Clinical Studies	Reference
Mesenchymal stromal cells-derived exosomes	Potential to improve neurological injury.	Understanding the effect of exosomes as mediators of the beneficial effects of cell therapy for stroke and traumatic brain injury.	[225]
Human hepatocellular carcinoma exosomes	Important role in the diagnosis and therapy for tumors. Screening for biomarkers in early formation of chronic hepatitis and liver cancer.	Elucidation of signal pathways and their involvement in growth, metastasis, and angiogenesis; and of the significance of exosomes in the treatment of hepatocellular carcinoma.	[227]
Human serum exosomes	Symptomatic respiratory viral infections after lung transplantation.	Presence of lung self-antigens, 20S proteasome, and viral antigens implies that exosomes trigger chronic rejection.	[255]
Optogenetically engineered exosome system (EXPLOR)	Therapeutic carriers to deliver srikB (super-repressor IkB) to a therapeutic target used as sepsis model.	Amelioration of sepsis-induced organ injury and inhibition of secretion of proinflammatory cytokines.	[256]
Human placenta exosomes Maternal plasma exosomes	Therapeutic approach for gestational diabetes mellitus.	To reduce the negative impact of gestational diabetes mellitus.	[257]
Engineered tumor-derived exosomes	Therapeutic approach as anti-tumor agents.	Potentials usage in cancer immunotherapy.	[258]
Mouse adipose- derived mesenchymal stem cell exosomes	Novel therapeutic strategy for tissue injury.	Proteomic analysis of exosomes found more than 1000 protein groups with a number of biological functions, implying such exosomes might be valuable as potential therapeutic targets for tissue repair.	[259]
Mesenchymal stem cell-derived exosomes	Potential role in osteoarthritis regenerative medicine.	Elucidation of the inflammatory and multiple pathophysiological processes in the synovium, leading to the degradation of cartilage and bone. Potential role in cartilage repair and osteoarthritis therapy.	[260]
Vascular endothelial cells-derived exosomes Vascular smooth muscle cells-derived exosomes Several other cells-derived exosomes	Potential of exosomes in diagnosis, prognosis, and treatment of atherosclerosis.	Understanding of occurrence and development of cardiovascular diseases such as atherosclerosis.	[261]
Human urine exosomes	Potential of exosomes in diagnosis of nephropathies.	Characterization of proteolytically derived peptides that are essentially relevant to classify patients with nephropathies, cancers of the urinary tract.	[262]
Human urine exosomes	Potential of exosome gene expression assay as noninvasive test for prostate cancer.	Evaluation of a urinary diagnostic assay to help assess whether a prostate biopsy is warranted.	[263]
Human bone marrow mesenchymal stem cells-derived exosomes	Potential of exosome as treatment for severe COVID-19.	Understanding of the effect and capacity of exosomes to restore oxygenation, downregulate cytokine storm, and reconstitute immunity.	[264]
Human lungs epithelial cells-derived exosomes transduced with selected genes of the SARS-CoV-2	A new strategy to demonstrate that SARS-CoV-2 RNA-containing exosomes represent an indirect route of entry into cardiomyocytes.	Understanding a potential cardiac dysfunction produced via SARS-CoV-2 RNA containing exosomes, without the need for direct viral infection.	[265]
Bovine milk exosomes	An alternative strategy to load hydrophilic and lipophilic small molecules, and chemotherapeutic drugs into exosomes.	Potential drug delivery vehicle or nanocarrier for cancer treatment.	[266]
Mesenchymal stem/stromal cell-derived exosomes	May provide considerable advantages over their counterpart live cells, potentially reducing undesirable side effects including infusional toxicities.	Potential use in gene delivery, regenerative medicine, and immunomodulation.	[267]
Cultured third-molar pulp cell-derived exosomes	Potential of pulp-derived exosomes in combination with fibrin gel to fill dental hard tissues	Understanding the use exosomes in combination with fibrin gel in clinical translation towards improved cell-free regenerative endodontics.	[268]
Tumor-derived exosomes (TEXs)	Potential for circulating immune-related biomarkers, reflecting partially the genetic and molecular contents of the parent cancer cell.	Understanding how TEXs influence boost tumor growth, regulation of tumor neo-angiogenesis, premetastatic niche formation, and therapy resistance.	[269]
Human exosomes-based vaccines	Potential use of the S protein of the SARS-CoV, a type I transmembrane glycoprotein, incorporated into exosomes as antigen to be used in vaccines to induce high levels of neutralizing antibodies.	Manufacturing of highly immunogenic SARS-S-based vaccines. Additionally, potential use in inhibiting tumor growth.	[270]
Exosomal noncoding RNA in glioma	Potential of exosomes as carriers of bioactive molecules into the brain. Holds great promise in diagnostics and therapy. Noncoding RNAs of exosomes can be modulators of numerous hallmarks of glioma.	Understanding the function of exosomal noncoding RNAs in cell-to-cell communication in the tumor microenvironment, tumor proliferation, invasion, angiogenesis, immune-scape, and treatment resistance.	[271]
Human exosomes in cardiovascular diseases	Potential of exosomes as more effective intervention targets on ischemic heart disease. The presence of exosomes in plaque tissue, ischemic heart, and peripheral blood can be potential biomarkers for early diagnosis and prognosis of cardiovascular diseases.	Understanding the participation of exosomes in the evolution of ischemic heart disease, including their role in endothelial dysfunction, lipid deposition, atheromatous plaque formation and rupture, ischemia-reperfusion, and heart failure.	[272]
Human urine exosomes	Potential source of biomarkers, pathogenic molecules, and therapeutic biologics in kidney diseases or disorders.	Understanding the role of exosomes in pathogenic mechanisms, biomarker discovery, and therapeutics of various kidney diseases, particularly lupus nephritis, acute kidney injury, diabetic nephropathy, renal fibrosis, kidney transplantation, and renal carcinoma.	[273]
Exosomes in inflammatory processes	Potential of exosome’s cargo, such as the novel SLC22A5 transport protein, to serve as useful biomarker of inflammatory processes.	Understanding the significance of exosomes as carriers of inflammatory mediators involved in human pathologies.	[274]
Human saliva exosomes from HIV-positive people	Platform to demonstrate that isolated saliva exosomes from HIV-positive individuals promote Kaposi’s sarcoma-associated herpes virus (KSHV) infectivity in human oral epithelial cells.	Understanding how the trans-activation response element (TAR) RNA in HIV-associated exosomes contribute to enhancing KSHV infectivity through the epidermal growth factor receptor (EGFR).	[275]
Human semen exosomes	Potential of exosome’s cargo containing molecular fingerprints for a non-invasive diagnosis of prostate cancer.	Understanding the role of semen exosomes in prostate cancer diagnosis, and as possible agents for enhancing the transmission of sexual diseases.	[276]
Human serum exosomes	Potential role of some miRNAs extracted from serum exosomes of Parkinson’s disease patients to serve as biomarkers.	Understanding the significance of the expression levels of miR19b, miR24, and miR195 as biomarkers of Parkinson’s disease.	[277]
Human milk exosomes	A complementary strategy to deliver more functional insights of human milk.	Providing an enhanced immunological and micronutrient profile of human milk, with significant relevance to breast milk quality and the health of the mother and infant.	[278]
Human umbilical cord exosomes	Potential role of exosomes derived from Akt-modified human umbilical cord mesenchymal stem cells as therapy for improving cardiac regeneration.	Understanding the role of why exosomes obtained from Akt-modified umbilical cord mesenchymal stem cells are more effective as therapy in myocardial infarction by promoting angiogenesis via activating platelet-derived growth factor D.	[279]
Human bronchoalveolar lavage fluid exosomes	Potential role of miRNA from exosomes with proinflammatory signatures in asthma and in allergic airway diseases.	Understanding the role of miRNA obtained from bronchoalveolar lavage fluid exosomes. Particularly microRNAs miR-24 and miR-27, which can modulate gene programming and promote inflammation.	[280]
Human cerebrospinal fluid and plasma exosomes	Potential use of alpha-synuclein and tau proteins obtained from central nervous system-derived exosomes that can efflux into blood can be used as biomarkers of Parkinson’s disease and other neurodegenerative diseases. Exosomes can also serve as carriers of therapeutic substances for diseases of the central nervous system.	Understanding the role of the content of exosomes derived from the central nervous system in Parkinson’s disease. Exosomes can carry and spread toxic alpha-synuclein between cells and induce apoptosis.	[281]
Human induced pluripotent stem cell-derived neuronal exosomes	Potential use as a tool to assay the capacity of exosomes to influence neuronal and circuit development. Control exosomes rescue neurodevelopmental defects in a model of Rett syndrome.	Understanding the role of exosomes in the development of neural circuits, the increase in neurogenesis, and the promotion of cell proliferation and neural differentiation.	[282]
Blood or cerebrospinal fluid-derived circulating circular exosomal RNAs	Potential use of circulating exosomal circular RNAs (circRNAs) as biomarkers for the early detection and diagnosis of neuropsychiatric disorders.	Understanding the role of closed-loop structure circular RNAs, a novel class of non-coding RNA (ncRNA), in mental diseases. Some studies show that circRNAs possess regulatory potential as “sponges” for target microRNAs (miRNAs) and RNA binding proteins.	[283]
Ovarian cancer cells-derived exosomes	Potential use of exosomal proteins and lipids in the early diagnosis of ovarian cancer.	Understanding the role several lipid species and proteins, which significantly differ in cancer derived exosomes when compared to those from ovarian surface epithelial cells.	[284]
